# A qualitative exploration of the unmet information needs of Chinese advanced cancer patients and their informal caregivers

**DOI:** 10.1186/s12904-021-00774-7

**Published:** 2021-06-07

**Authors:** Tao Wang, Alex Molassiotis, Betty Pui Man Chung, Si-Lin Zheng, Hou-Qiang Huang, Jing-Yu (Benjamin) Tan

**Affiliations:** 1 College of Nursing and Midwifery Brisbane Centre, Charles Darwin University, Brisbane, Australia; 2 School of Nursing, The Hong Kong Polytechnic University, Hung Hom, Hong Kong, Hong Kong; 3 Department of Nursing, The Affiliated Hospital of Southwest Medical University, Luzhou, People’s Republic of China

**Keywords:** Information needs, Cancer survivors, Caregivers, China

## Abstract

**Background:**

Studies in the West have demonstrated that appropriate informational support is a vital component of cancer care, with positive effects on both patients and their informal caregivers. Since little is known about the information needs of advanced cancer patients and informal caregivers in China, where ‘silence as virtue’ is much more valued and the communication style is less open, this study was therefore conducted to elaborate the information needs of advanced cancer patients and informal caregivers as well as to explore their perceptions and experiences regarding their unmet information needs in the Chinese context.

**Methods:**

This sub-study of a previous cross-sectional survey utilized a qualitative descriptive study design. The approach involved semi-structured interviews that followed an interview guide to collect data. Eligible participants were the advanced cancer patients and informal caregivers who had participated in the previous cross-sectional survey and reported unmet information needs. Each interview was audio-recorded and transcribed verbatim. Descriptive content analysis was used to analyze the data.

**Results:**

Seventeen advanced cancer patients and 15 informal caregivers with unmet information needs participated in the semi-structured interviews, with ages ranging from 32 to 63 years old for patients and from 32 to 70 for informal caregivers. Four categories were extracted from the interviews with the patients and caregivers: (1) types of unmet information needs; (2) reasons for information needs not being met; (3) preferences for the provision of information; and (4) meaning and role of information. Each category had two to four sub-categories for both the patients and the caregivers, which were similar but not completely the same.

**Conclusion:**

The findings indicated that the provision of appropriate information could promote informed decision-making and greater satisfaction with treatment options, reductions in psychological disturbances, and enhanced confidence and ability in self-management and capacity in caregiving. Moreover, information on Traditional Chinese Medicine and food therapy should be increased, particularly for patients at the follow-up stage, while the amount of information on prognosis should be flexible as it could increase patients’ and caregivers’ psychological burden. Healthcare professionals were the most preferred information provider, although their heavy workload resulted in time constraints. In this case, they should provide information to patients and caregivers together as a ‘whole unit.’ At the same time, the value of separate conversations should also be recognized as some caregivers preferred to conceal unpleasant information from the patient.

## Introduction

According to the National Central Cancer Registry of China [1], the incidence of cancer in China is about 278.07 per 100,000. In 2018, there were around 4.3 million new cases of cancer and 2.9 million new cancer deaths in China [2]. Cancer has become one of the leading causes of death in China [3]. Patients with cancer often experience more feelings of shock, fear, uncertainty, and loss of control than those with many other diagnoses [4], and appropriate informational support is vital in helping cancer patients cope with negative feelings and conditions [5, 6]. Other positive effects of adequate and appropriate informational support have also been identified in several studies, including increased involvement in the decision-making process [7], increased satisfaction with treatment choices [8], and better quality of life [9]. However, cancer patients’ information needs are often not met in clinical practice, and the information needs of Chinese advanced cancer patients have been documented as a frequently reported unmet care need [10].

The concept of viewing patients with life-limiting diseases and their informal caregivers as ‘a whole unit’ has been emphasized in palliative care, and informal caregivers are often regarded as potential ‘co-clients’ or ‘co-users’ of care [11]. Informal caregivers are an important resource for the clinical care of cancer patients and as such they usually undertake many unfamiliar and uncompensated caregiving tasks, such as physical care [12], emotional care [13], and medical treatment monitoring [14]. Cancer patients at an advanced stage often experience more complex symptoms than those at an early stage, which can further increase informal caregivers’ caregiving tasks [15]. Increased caregiving tasks may subsequently increase caregivers’ need for substantial information to improve their caregiving competencies and to deal with uncertainties and problems during the caregiving process [16].

Over the past few decades, extensive research efforts have been devoted to the information needs of cancer patients [17]. However, most of the studies were conducted in Western countries, and little is known about Chinese cancer patients. Different from Western cultures, where the concept of ‘open communication’ ([18]: p.150) is much more valued, the communication style of Chinese people is often ‘less open and more indirect’ ([18]: p.149]) as silence is viewed as a virtue in many Asian countries, including China, due to the influences of Confucianism. Moreover, Chinese people prefer to share ‘happiness’ rather than ‘worries’ (报喜不报忧) [19], which may induce healthcare professionals and informal caregivers to withhold information from patients. Considering that people’s perceptions and cognitions are formed by the culture and society to which they belong, the need for certain information and the reasons for this need among cancer patients in Eastern cultures may differ from those in Western cultures [18]. Additionally, although appropriate and timely information is an important facilitator for successful caregiving [20] and the concept of ‘patient-and-family-centered care’ has been emphasized by the World Health Organization [21], studies that have evaluated the specific information needs of informal caregivers are still relatively rare [22].

According to a previous cross-sectional survey [23], information needs was a frequently reported need for both Chinese advanced cancer patients and their informal caregivers. This finding was different from the findings of a previous systematic review [10], which analyzed 50 international studies (86% were studies conducted outside Mainland China) and showed that physical and psychological needs were much more prevalent than information needs, particularly for patients. Although information needs was identified as a frequently reported unmet need, little knowledge could be drawn from the cross-sectional survey due to the limited number of items on information needs assessment included in the questionnaire [23]. The current qualitative study was therefore designed and conducted subsequently to extend this prior research, with the aim of further elaborating the patients’ and informal caregivers’ unmet information needs as well as exploring their perceptions and experiences regarding their unmet information needs in the Chinese context.

## Methods

### Study design and setting

The current study was a sub-study of a previous cross-sectional survey [23]. In the cross-sectional survey, 419 patient-informal caregiver dyads were recruited, from April 2018 to January 2019, to identify the palliative care needs of patients and their informal caregivers. Information needs was quantified as a frequently reported unmet need in the survey by both patients and caregivers. Given that very limited knowledge about information needs could be drawn from the survey, a follow-up qualitative study was subsequently conducted to further elaborate and explore the perceptions and experiences of the patients and caregivers in relation to their unmet information needs.

A qualitative descriptive design is often used to achieve a pure description without following a specific qualitative research tradition [24]. The qualitative description design has been promoted as a well-developed method to provide a ‘comprehensive summary of an event in the everyday terms of those events’ ([25]: p.336). Considering the research aims of this sub-study, a qualitative descriptive design was employed. Ethical approvals were granted by the Human Subjects Ethics Sub-Committee at The Hong Kong Polytechnic University and The Affiliated Hospital of North Sichuan Medical College. All methods in this study were carried out in accordance with relevant guidelines and regulations.

### Participants

Although all the participants in the cross-sectional survey were informed that they may be approached again after the survey to participate in a follow-up qualitative interview, further face-to-face explanations of the qualitative sub-study process and research aims were given again to potential participants after the survey to invite them to participate. Those who were willing to participate were required to sign a consent form for this qualitative sub-study. Given that this qualitative sub-study was an extension of prior research [23], purposive sampling was used to recruit participants from early February 2019 to the end of March 2019. The participants, including patients and informal caregivers, were approached if they: (1) had completed the cross-sectional survey [23]; (2) reported unmet information needs (for patients, this was determined using the Problems and Needs in Palliative Care-short version questionnaire, with the answer of ‘yes, more’ for the question ‘Do you want professional attention for this?’; for caregivers, this was determined using the Comprehensive Needs Assessment Tool in Cancer for Caregivers, with answers of moderate or high levels of information needs) [23]; (3) were physically capable of participating in an interview lasting 30 to 60 min; and (4) agreed to participate in the sub-study and signed a written informed consent form. Seventeen patients and 15 informal caregivers were recruited based on data saturation [26]. All of the participants who were approached to take part in the qualitative interviews did so, yielding a response rate of 100%.

### Data collection

A semi-structured interview was used to collect data since it combined the strengths and eliminated the weaknesses of structured and unstructured interviews, and it is currently the most commonly used approach for qualitative data collection in healthcare research [27]. Semi-structured interviews enable an in-depth understanding of the participants’ perceptions and/or experiences of one focused research topic [27, 28]. The interview guides used in this study were comprehensively developed based on the expertise of the research group, a series of previous qualitative studies and mixed-methods studies that investigated patients’ and/or informal caregivers’ views and/or experiences in relation to information needs [29–31], and validation through pilot interviews. Six key questions were included in the interview guide to explore the following areas: the information that the participants desired to learn (what and why); the time that the participants needed the information most (when); the participants’ current provision of information (what was provided, who provided it, where, and in what format); the participants’ feelings about the information that they received; factors or reasons that resulted in the participants’ unmet information needs; and the participants’ perceptions regarding how the information could affect them if they received sufficient information. The patients and caregivers were interviewed separately at the study hospital by the first author (TW), who was a doctoral researcher at that time. Prior to the commencement of the interviews, the doctoral researcher had completed qualitative training and had fully practiced her interview skills through a few pilot interviews with the guidance of an experienced qualitative researcher (the third author, BPMC). Before each interview, a pre-designed form was used to collect the participant’s demographic and medical information. Each interview was audio-recorded.

### Data analysis

Qualitative content analysis was employed to analyze the data following the ‘four phases of category development’ method [32], which includes ‘initialization,’ ‘construction,’ ‘rectification,’ and ‘finalization.’ In the ‘initialization’ phase, the transcribed data were read and re-read many times to become familiar with the data. The phrases, sentences, and/or paragraphs that were relevant to the research questions were highlighted as meaning units and codes. In the ‘construction’ phase, the initial codes were classified and compared regarding their similarities and differences, and then they were assigned to different groups based on the research questions and on the principle of ‘mutual exclusivity.’ ‘Rectification’ was then conducted to further modify the categories and sub-categories by reappraising the analysis process at either the coding process level or the whole dataset level. The final phase was ‘finalization,’ which involved using representative data (i.e., quotes) that were extracted from the transcriptions to support each category and/or sub-category and to report the results of the analysis.

Several strategies were used to achieve rigor and trustworthiness in this study. All transcripts were checked against the original audio-taped records to ensure accuracy. Separate coding was used for some transcripts (i.e., coded independently by the first author [TW] and two other experienced qualitative researchers with PhD degrees) to develop the coding structure. The first author (TW) then completed the rest of the coding based on the coding structure. Ongoing discussions among group members were performed during the whole data analysis process. Two bilingual translators translated the quotes to ensure the equivalence of the participants’ descriptions between different languages (i.e., English and Mandarin), and a third party was involved when inconsistency existed. The identified categories and sub-categories were sent back to several participants to check whether these categories indicated their perceptions and experiences. Trust and rapport with the participants were built prior to the interviews as the interviewer (TW) had stayed at the study site for 10 months due to the previous survey.

## Findings

### Characteristics of the participants

The characteristics of the participants are presented in Tables 1 and 2.

**Table 1:** Characteristics of the advanced cancer patients (*n* = 17)

Patients	Gender	Age	Diagnosis	Cancer Stage	Education	Marital Status	Occupation	Religion
P1	Male	56	Nasopharyngeal carcinoma	III	Illiterate	Married	Manual worker	No
P2	Male	51	Lung cancer	IV	Middle school	Married	Manual worker	No
P3	Female	55	Cervical cancer	IV	Primary school	Widowed	Manual worker	No
P4	Female	32	Cervical cancer	IV	High school	Divorced	Kindergarten teacher	No
P5	Female	36	Cervical cancer	III	High school	Married	Unemployed	No
P6	Male	48	Hepatic carcinoma	IV	Primary school	Married	Unemployed	No
P7	Male	60	Colorectal cancer	IV	High school	Married	Retired	No
P8	Female	45	Cervical cancer	III	Primary school	Married	Manual worker	No
P9	Female	43	Nasopharyngeal carcinoma	IV	Primary school	Married	Technician	No
P10	Male	63	Colorectal cancer	IV	Primary school	Married	Manual worker	No
P11	Female	43	Ovarian cancer	III	Primary school	Married	Self-employed	No
P12	Male	45	Lung cancer	IV	Primary school	Married	Manual worker	No
P13	Male	54	Esophageal cancer	IV	Middle school	Married	Self-employed	No
P14	Male	63	Esophageal cancer	IV	Primary school	Married	Manual worker	Buddhism
P15	Female	49	Lung cancer	IV	Primary school	Married	Manual worker	No
P16	Male	56	Lung cancer	IV	Middle school	Married	Unemployed	No
P17	Female	55	Lung cancer	IV	Primary school	Married	Manual worker	No

**Table 2:** Characteristics of the informal caregivers (*n* = 15)

Caregivers	Relationship with Patients^a^	Gender	Age	Education	Marital Status	Occupation	Religion	Patient’s Diagnosis	Patient’s Cancer Stage
C1	Son	Male	34	Primary school	Married	Unemployed	No	Lung cancer	IV
C2	Husband	Male	53	Middle school	Married	Self-employed	No	Lung cancer	IV
C3	Wife	Female	49	Middle school	Married	Manual worker	No	Lung cancer	IV
C4	Son	Male	35	High school	Married	Self-employed	No	Lung cancer	IV
C5	Son	Male	36	Middle school	Married	Manual worker	No	Lung cancer	III
C6	Wife	Female	53	Primary school	Married	Unemployed	No	Esophageal cancer	III
C7	Husband	Male	50	Primary school	Married	Unemployed	No	Cervical cancer	III
C8	Daughter	Female	33	High school	Married	Self-employed	No	Hepatic carcinoma	IV
C9	Wife	Female	46	Primary school	Married	Unemployed	No	Lung cancer	IV
C10	Wife	Female	52	Illiterate	Married	Manual worker	No	Esophageal cancer	IV
C11	Wife	Female	70	Illiterate	Married	Manual worker	No	Esophageal cancer	IV
C12	Wife	Female	70	Middle school	Married	Retired	No	Lung cancer	IV
C13	Husband	Male	73	Primary school	Married	Retired	No	Breast cancer	IV
C14	Son	Male	32	High school	Single	Unemployed	No	Gastric cancer	IV
C15	Wife	Female	57	Primary school	Married	Retired	Buddhism	Colorectal cancer	IV

Note: ^a^Relationship with patients means, for example, for C1, the informal caregiver is the son of the patient

### Perceived information needs of advanced cancer patients and their informal caregivers

Four categories were identified in the interviews with the patients and caregivers: (1) types of unmet information needs; (2) reasons for information needs not being met; (3) preferences for provision of information; and (4) meaning and role of information. Each category had two to four sub-categories for both the patients and the caregivers, which were similar but not entirely the same (see Table 3).

**Table 3:** Summary of the categories and sub-categories of the patients and caregivers

Categories	Sub-categories	
	**Patients**	**Informal Caregivers**
Types of unmet information needs	• Disease- and treatment-related information	• Disease- and treatment-related information
	• Daily life, particularly food therapy	• Caregiving-related information
	• Psychological and physical symptom management	• Psychological adjustment
	• Financial support	• Financial support
Reasons for information needs not being met	• Patient factors ○ Knowledge and beliefs ○ Poor health status ○ Low motivation: Fear	• Caregiver factors ○ Knowledge and beliefs ○ Low motivation: Fear
	• Healthcare professional factors ○ Have no time due to their heavy workload ○ Uncertain answers	• Healthcare professional factor ○ Have no time due to their heavy workload
	• Family/social support ○ Insufficient family support ○ Social isolation	• Family/social support ○ Insufficient family support
Preferences for provision of information	• Information provider: preferred healthcare professionals	• Information provider: preferred healthcare professionals
	• Information format: written information	• Information format: face-to-face conversations but preferred to hold back unpleasant information from the patient
	• Timing of information: changed and fluctuated throughout disease trajectory	• Timing of information: changed and fluctuated throughout disease trajectory
Meaning and role of information	• Self-management	• Being more prepared for caregiving role
	• Decision-making	• Decision-making and future planning
	• Hope for and chance of survival	• Hope for and chance of survival
	• Psychological impact	• Psychological impact

### Category 1: types of unmet information needs

Both the patients and the caregivers desired to obtain more information about disease conditions, progression and treatment, daily life issues (particularly food therapy), physical symptom management, self-emotion management, and information on insurance coverage and reimbursement.

### Disease- and treatment-related information

Disease- and treatment-related information was the most frequently reported unmet information need for both the patients (15/17) and the caregivers (15/15). Many of the patients and caregivers stated that they had inadequate information on disease conditions and progression and had a desire to learn more:

*“I want to know the conditions of my own disease. Are the conditions stable or anything worse? Is the tumor size bigger than before?”* (P17, Female, 55-year-old, Lung cancer, Stage IV)

*“For our family members, the thing that I mainly want to know is about his disease conditions. For example, if the disease is under control or not, or, if anything had become worse? Those are what I want to know.”* (C4, Son, 35-year-old, Patient with lung cancer)

Patients and caregivers also indicated that they could not obtain adequate treatment-related information from healthcare professionals:

*“Almost no, I can say I learn nothing [about the chemotherapy regimens]. This time, I was transferred to another doctor. I told the new doctor that I had serious vomiting, and I didn’t want to continue [the chemotherapy]. After that, the doctor suggested I use another regimen. But she only mentioned little about this, didn’t tell me any details.”* (P3, Female, 55-year-old, Cervical cancer, Stage IV)

*“Currently, we learn very little information about continuing the treatment regimens. At least, we learn little from the doctors.”* (C6, Wife, 53-year-old, Patient with esophageal cancer)

Given that Traditional Chinese Medicine (TCM) plays an important role in Chinese culture, information about TCM was one commonly reported type of treatment-related information that patients preferred to receive, particularly patients at the follow-up stage who were not satisfied with Western medicine-type treatment and those who had suffered from too many side effects from Western treatment:

*“We have visited the hospital many times, and we strictly followed the instructions of the doctors. I have received chemotherapy six times, oh, should be seven times. I cannot stand it anymore. I don’t want to continue the chemotherapy. He [the doctor] told me that I needed to take six times of chemotherapy, but he said this was a kind of…targeted medicine, so I need to actually take twelve times as two times together were counted as one time. There were actually no positive changes in the tumor size in the lung, no significant changes after the chemotherapy. Besides, the chemotherapy brought a lot of undesirable sufferings to me. So, I don’t want to receive the chemotherapy anymore. I plan to use traditional Chinese herbs to promote the recovery of my body. I want to learn some information about traditional Chinese herbs.”* (P7, Male, 60-year-old, Colorectal cancer [metastasized to the lung], Stage IV)

### Daily life, particularly food therapy

Around half of the patients (7/17) and caregivers (8/15) sought information on daily life. Given the belief in and popularity of food therapy in Chinese culture, diet was mentioned most frequently by both the patients and the caregivers:

*“Food therapy, you know, there are a lot of food remedies. When I was healthy, I usually made Chinese soup, putting some herbs, for example ‘Huang qi,’ ‘angelica’ in the soup to improve our health. However, now I am not sure if I can eat those herbs with this disease. For diet, now, what should I do, what food can I eat and what can I not eat; what kind of food is bad for my disease, and what kind of food is good for recovery from the disease? Those are what I want to know.”* (P5, Female, 36-year-old, Cervical cancer, Stage III)

*“I think that the hospital should provide some information to our family members, like, what we should do at home after discharge and information about diet at home for the patients; they need not only biomedical therapies but also food therapies. Yes, those kinds of information you can provide [to us].”* (C8, Daughter, 33-year-old, Patient with hepatic carcinoma)

### Physical and psychological symptom management

Although all the patients were advanced stage cancer patients, they were still receiving active anticancer treatment, particularly chemotherapy, which resulted in some chemotherapy-induced side effects. Several of the patients and caregivers indicated that they wanted to learn more about physical symptom management, such as pain and chemotherapy-induced nausea and vomiting:

*“I want to know whether there are any other approaches that I could use to relieve my pain. I don’t want to depend on the painkiller only.”* (P17, Female, 55-year-old, Lung cancer, Stage IV)

*“Every time after he received the chemotherapy, he suffered from serious vomiting. So, I want to know how to relieve this symptom, to decrease his distress and make him comfortable.”* (C3, Wife, 49-year-old, Patient with lung cancer)

Moreover, given that suffering from cancer was a stressor for patients, patients also needed more information about psychological adjustment to maintain their own mental health:

*“I am not sure whether these methods [for emotions adjustment] were right or not, but at least I think my emotional status is okay. So, I think the methods that I used are okay. Of course, it would be better if you [healthcare professionals] could teach us some methods to adjust our emotions.”* (P1, Male, 56-year-old, Nasopharyngeal cancer, Stage III)

Caregivers also wanted to learn more about self-emotion management; however, their purpose was to conceal their own negative emotions and show more positive ones in front of the patient:

*“As his family, I should keep happy in front of the patient to decrease his psychological burden. Because once we are not happy, the patient will subsequently feel more stressed. So, even if I am sad, I need pretend to be happy in front of the patient to let him have a good mood, but how can I do that? I want to know.”* (C14, Son, 32-year-old, Patient with gastric cancer)

### Financial support

Most of the interviewed patients had basic national insurance, which partly alleviated their financial burden. However, several of the patients and caregivers reported that they had learned very little about national insurance coverage and wanted to learn more, particularly about the coverage offered and reimbursement:

*“Every time after I received the chemotherapy, I needed to inject some medicines to increase the number of white blood cells and red blood cells. The price was more than one thousand for each injection. It seems that it was not covered by the insurance and we cannot claim any reimbursement. I want to know if it was really not covered. I indeed learned little in terms of the insurance reimbursement.”* (P2, Male, 51-year-old, Lung cancer, Stage IV)

*“Another thing that I want to learn is about medical reimbursement. We are not locals, so we are not clear about it. It seems that the reimbursement proportion is 65% if we claim it in Nanchong city. But I am not sure if we can claim more reimbursement when we come back to our city.”* (C3, Wife, 49-year-old, Patient with lung cancer).

### Category 2: reasons for information needs not being met

The heavy workload of healthcare professionals, poor literacy and beliefs of individuals, fear of unpleasant information, and insufficient family support were common reasons for the information needs of patients and caregivers not being met. Poor health status and social isolation due to the disease were two other unique reasons for the patients.

### Healthcare professional factors

Most of the patients (13/17) and nearly half of the caregivers (7/15) said that healthcare professionals were too busy to provide them with detailed information. It was one of the most important reasons that their information needs were not being met:

*“Doctors are too busy to talk with us. You know, there are so many patients that the doctors cannot detail everything to us. The doctors can only tell us the most important things, like the condition of the disease.”* (P1, Male, 56-year-old, Nasopharyngeal cancer, Stage III)

Some of the caregivers also shared their experiences of being rushed when seeking information from doctors:

*“They [doctors] are busy and they don’t have enough time to talk with us unless it is an urgent issue. Every time when I saw they were busy I did not ask them for information. Sometimes when I got the chance to ask, I felt sorry for occupying too much time as many other patients and families are waiting for the doctor. So, I cannot get too much information from the doctor, just a few sentences.”* (C3, Wife, 49-year-old, Patient with lung cancer)

In addition, uncertain answers from doctors was another reason for the patients’ unmet information needs:

*“Even if you ask the doctor, he or she won’t give you a definite answer. [He or she] won’t tell you to what extent the drug might help with your condition. Not 100 per cent...even 80 to 90 per cent.... He won’t tell! How and what could you expect me to get such information [from the doctor]? He has no idea, me neither!”* (P15, Female, 49-year-old, Lung cancer, Stage IV)

### Individual factors

#### Knowledge and beliefs

Both the patients and the caregivers indicated that their poor literacy to some extent limited their ability to seek and understand information when it was given:

*“For many things, I know it is important. However, I don’t know how to learn it more or in detail because of my poor education level. For example, many people know how to search for information online, but I don’t know how to do that. I usually cannot find the information that I want to know due to my poor literacy.”* (P8, Female, 45-year-old, Cervical cancer, Stage III)

*“I am illiterate. I have never received any formal education. So, sometimes I can’t understand the words that the doctor tells me unless he or she explains it to me several times.”* (C11, Wife, 70-year-old, Patient with esophageal cancer)

Although information about daily life such as diet was reported as a type of information that the patients and caregivers wanted to obtain, they did not actively seek this kind of information. They viewed daily life information as less important compared with information about the disease and its treatment. The doctors’ priority was ‘curing’ the disease rather than talking about daily life issues:

*“Just like diet, it is a tiny issue. If the doctors were really busy and had no time, I would not disturb and occupy the time of doctors due to this kind of tiny issue.”* (P5, Female, 36-year-old, Cervical cancer, Stage III)

*“When my husband feels unwell, I visit and report it to the doctor. I would feel relaxed if the doctor told me that the patient’s condition was okay. While for daily diet, I think it is not that urgent, I seldom ask for this kind of information from doctors.”* (C6, Wife, 53-year-old, Patient with esophageal cancer)

#### Poor health status of patients

The patients usually experienced unpleasant side effects such as nausea and vomiting after receiving chemotherapy. They were therefore not physically well enough to seek information until they had recovered from the side effects:

*“I am old now and I feel really unwell due to the nausea and vomiting after the chemotherapy. The poor fitness makes me not seek information.”* (P3, Female, 55-year-old, Cervical cancer, Stage IV)

#### Low motivation: fear

Several of the patients and caregivers expressed psychological conflicts in information seeking. On the one hand, they wanted to obtain information, but on the other hand, they avoided asking for information as they were afraid of hearing bad news. Fear therefore had reduced their motivation to ask for information:

*“Sometimes…I feel that it would be worse if I learned more. Instead, learning little might be better. For instance, if my doctor told me that the review results were good, of course, I would be happy. However, if the results were not good, I would have totally different emotions. So, sometimes I feel conflicted in terms of learning information. I want to learn something but fear that it is bad information. So just let it go, not seek information actively, learning little might be better.”* (P16, Male, 56-year-old, Lung cancer, Stage IV)

*“On the one hand, I really want to learn something about him, but on the other hand, I am afraid of knowing the truth. If you ask me whether I want to learn more information, of course, my answer is yes. But I am not willing to learn information by myself. I ask his young brother and our son-in-law to learn the information, and then I can learn something from them if I want to.”* (C15, Wife, 57-year-old, Patient with colorectal cancer)

### Family/social support factors

Several of the caregivers (all female) reported that they were the only person who was available to take care of the patient. Given the traditional gender roles in Chinese culture, females usually attend to housework. The caregivers not only had to look after the patient but also had to take care of domestic affairs. The heavy caregiving burden left them with no time or energy to seek information:

*“I am the only person who is taking care of him and I have to stay with him due to the transfusion, which makes me have no time to seek information.”* (C6, Wife, 53-year-old, Patient with esophageal cancer)

*“Sometimes, my wife learns some information for me; however, to be honest, she has no time to seek more information. We have only one child and she has her job, so it is my wife who does everything for me as well as the housework, I know she is burned out.”* (P6, Male, 48-year-old, Hepatic cancer, Stage IV)

Social isolation after the cancer diagnosis was also experienced by some of the patients, which also reduced their chances of obtaining information from others:

*“I am not an outgoing person. Since the diagnosis of cancer, I feel that I am further isolated from others, so I am not willing to communicate with others anymore. For example, when I come back home from hospital, I usually walk around alone and seldom have a chat with others. Actually, if I could have a chat with others, maybe I could obtain some information via chatting.”* (P14, Male, 63-year-old, Esophageal cancer, Stage IV)

### Category 3: preferences for provision of information

Both the patients and the caregivers preferred to get information from healthcare professionals. For most of the patients, they preferred written information, while for caregivers, face-to-face conversations was the most commonly mentioned format. The fluctuation of information needs throughout the disease trajectory was similar for the patients and caregivers—information on disease condition and treatment at the stage of diagnosis, daily care information, continuing treatment information after the completion of chemotherapy, and treatment information at the time of recurrence or of deterioration.

### Information provider

Healthcare professionals, particularly doctors, were regarded as the ideal information providers by both the patients and the caregivers. The information provided by healthcare professionals was regarded as much more reliable and believable than that from other sources:

*“Of course, I hope to learn some better information. There is too much information [outside] about diet, I cannot try each kind of food. If the doctors can tell me something about diet, it will be much better as I believe the doctors. Maybe the information provided by doctors is not 100% effective, but I think it is at least safe.”* (P17, Female, 55-year-old, Lung cancer, Stage IV)

*“[I want to learn information from doctors] because doctors are authorities and professionals. Their words are much more reliable and believable.”* (C1, Son, 34-year-old, Patient with lung cancer)

### Information format

The patients most commonly mentioned written information, such as printed materials, as written information helped them to overcome the problem of poor memory, allowing them to read the information at their own convenience:

*“I hope [healthcare professionals] can give us some printed materials, printing it on paper. On this occasion, we can take the booklets back home and read.”* (P1, Male, 56-year-old, Nasopharyngeal cancer, Stage III)

*“I mean, give us some printed materials at the time of discharge such as daily life care at home. We can read it at home as I will forget it soon if you tell me through an oral conversation.”* (P9, Female, 43-year-old, Nasopharyngeal carcinoma, Stage IV)

The caregivers most commonly mentioned face-to-face conversations with healthcare professionals as their preferred format, with some expressing concern about bad news being revealed to the patient. They preferred to hold back unpleasant information from the patient during a conversation in order to minimize the patient’s psychological burden:

*“For example, we can sit down and discuss together with the doctors [about the treatment regimens]. However, for information about disease conditions, particularly bad information, the doctors had better tell and discuss with our families only in the doctor’s office [and exclude the patient from the discussion, particularly for bad information].”* (C4, Son, 35-year-old, Patient with lung cancer)

### Timing of information

The information needs of both the patients and the caregivers were not static but changed and fluctuated throughout the disease trajectory. The fluctuating need for information by the patients and caregivers was similar to the progression of the disease. At the time of diagnosis, the patients and caregivers had a substantial need for information about their condition and/or treatment of the disease:

*“[When it was diagnosed,] I didn’t feel any pain or uncomfortable, so I thought it was not a very serious disease. At that time, the cancer actually had metastasized to my brain, but it didn’t metastasize to the bones, so I had no pain. Except for the symptom of hand twitching, I didn’t think I was a patient. So, I didn’t believe that I suffered from cancer, and I visited many hospitals including hospitals in Chengdu, and the diagnosis of cancer was confirmed finally. After that, I began to seek treatment information actively, I really wanted to find some information on treatment therapies, for example, if there were any effective medicines that I could use to ‘cure’ my disease.”* (P12, Male, 45-year-old, Lung cancer, Stage IV)

*“I had never learned about cancer before, so I did not know what I should do when one of my family members suddenly suffered from cancer. I totally knew nothing. So, at the time when he was diagnosed with cancer, I really wanted to learn information about his disease like his disease conditions, whether it was curable, and how to cure his disease. I wanted to try my best to cure his disease.”* (C1, Son, 34-year-old, Patient with lung cancer)

After the patients completed active treatment, their information needs and those of their informal caregivers shifted to daily life care and/or continuing treatment at home to maintain the treatment effects:

*“Now, I have completed the treatment in the hospital. What I care about now is how to maintain the treatment effects and how to delay the progress of the disease through diet and daily care at home.”* (P6, Male, 48-year-old, Hepatic cancer, Stage IV)

*“This is the last chemotherapy, then it will be the follow-up. I will stay at home for most of the time, so I am thinking about how to take care of her better. To maintain the treatment effects, what should I do during the time at home?”* (C7, Husband, 50-year-old, Patient with cervical cancer)

With the progression of the disease, the need for treatment information increased again, especially when the patient’s condition became worse or the disease recurred:

*“Because the disease recurred, and chemotherapy didn’t make sense anymore, the tumor size is bigger than before.”* (P15, Female, 49-year-old, Lung cancer, Stage IV)

*“Now it has metastasized to the whole body and the chemotherapy has not made too many positive effects. On this occasion, I want to know more treatment information.”* (C2, Husband, 53-year-old, Patient with lung cancer)

### Category 4: meaning and role of information

Both the patients and the caregivers indicated that information played an important role in their efforts to cope with cancer. For most of the patients and caregivers, adequate information, such as treatment information, provided them with references when making medical decisions, increased their hope for and perhaps also chance of survival, and affected them psychologically, both positively and negatively. In addition, adequate information could assist the patients in their self-management and prepared caregivers for their role.

### Decision-making

“Getting more information would benefit decision-making” was mentioned by both the patients and the caregivers. They indicated that adequate information could help them make a medical decision wisely and comprehensively by understanding the potential treatment effects and side effects of the treatment regimens, as well as considering their own economic situation:

*“Learning more information will benefit my disease as I will know what kind of drug is good, what is bad for my body, and what has fewer side effects. The chemotherapy drugs that I currently use brought serious side effects to me, especially vomiting. If I could learn the chemotherapy drugs clearly, maybe I will choose the drugs with fewer side effects.”* (P2, Male, 51-year-old, Lung cancer, Stage IV)

*“If the doctor can provide us with more treatment regimens, like the advantages, side effects, potential effects, and expense of each regimen, it would help us have a better understanding of the regimen and then make a better decision.”* (C5, Son, 36-year-old, Patient with lung cancer)

The caregivers also stated that gaining adequate information about their loved one’s condition could help them plan for the future as well:

*“Some pieces of information regarding the [cancer] condition and prognosis would...[at least] enable us to have some preparations and plans for the things ahead…[at least] with some clues for some future planning...yeah...just such sorts of feelings.”* (C14, Son, 32-year-old, Patient with gastric cancer)

### Hope for and chance of survival

Both the patients and the caregivers indicated that having adequate information meant a greater chance and more hope for survival. Cancer posed a financial burden on most of the families with cancer patients. Families experiencing economic hardship sometimes had to forego treatments for financial reasons. Having more information on financial support, such as insurance coverage and reimbursement, could enable them to afford more and longer treatments, thus increasing their hope for survival:

*“If I had learned the information about reimbursement of the medical insurance, maybe I would use some medications with better effects and claim more for reimbursement. The reimbursement could support me to get more treatments, and having more treatments means more chances to survive.”* (P3, Female, 55-year-old, Cervical cancer, Stage IV)

*“Of course, I wish to know more about healthcare insurance. The healthcare insurance can, more or less, reimburse some coins...as you know, the cancer and related treatments really cost us a big sum of money. Some reimbursement [from the healthcare insurance] would at least enable us to afford a longer treatment with greater hope.”* (C3, Wife, 49-year-old, Patient with lung cancer)

Many other patients and caregivers reported that learning more about treatment regimens meant that they would have more options and the chance to try different treatments, increasing their chance of and hope for survival:

*“If we were informed of information on treatment regimens, we could select one to treat the disease continually. Only when we try more, the patient may have the chance to survive. So, learning more information on treatment regimens may enable us have a chance to choose the treatment and more chances for the patient to survive.”* (C2, Husband, 53-year-old, Patient with lung cancer)

*“Now I learn very little about cancer treatment regimens; if I could learn more, it would bring benefits. I would be much more confident as I could have more choices and more chances for us to have a try.”* (P7, Male, 60-year-old, Colorectal cancer, Stage IV)

### Psychological impact

Adequate information had both a positive and a negative psychological effect on the patients and caregivers. They stated that gaining more information would help them to understand their/the patients’ disease conditions better and more clearly, which could reduce their psychological pressure and increase their certainty and ease:

*“Information may influence our moods and psychological status. Knowing more information can help me learn my disease conditions better and know what I should do in daily life, which can enable me to feel certain. If I knew all this information clearly, I would not feel confused about my condition every day, such as ‘what is this?’ and ‘what should I do?’ I would feel much easier and relaxed.”* (P2, Male, 51-year-old, Lung cancer, Stage IV)

*“If I could learn more information, I would have a clear understanding about the patient’s disease condition. For treatment regimens, if I learned that the current treatment regimen is appropriate for him, of course, for us…no matter the patient or our family members, we will feel less stressed.”* (C4, Son, 35-year-old, Patient with lung cancer)

Moreover, being informed with information, particularly disease-related information, would reduce their doubts and uncertainties:

*“Having information versus having no information, I think learning more information would make me feel certain. If I know nothing, there would be a doubt in my mind.”* (C12, Wife, 70-year-old, Patient with lung cancer)

*“Generally, learning more information would make me feel at ease in many aspects, including the psychological aspect, because there would be no doubt in my mind. If I learn little, I will doubt my disease conditions all the time, for example, if the disease has become better or not. Knowing sufficient information would let me have the feeling of certainty.”* (P12, Male, 45-year-old, Lung cancer, Stage IV)

Negative impacts were also mentioned by the patients and caregivers. They believed that too much information might increase their psychological burden:

*“Learning too much, feeling much more upset. I, therefore, decide to follow the doctors and listen to them.”* (P5, Female, 36-year-old, Cervical cancer, Stage III)

*“I feel…if I learned more and clearly, I would be much more upset and sad. So now I don’t want to know too much. Sometimes, I don’t even know how to continue my life if he passed away.”* (C15, Wife, 57-year-old, Patient with colorectal cancer)

### Self-management

Over one-third of the patients expressed the view that knowing information, particularly daily life information like diet, could be a way of assisting them in self-management. Getting information on daily life could promote their adjustment to a different lifestyle and improve their capacity for self-management:

*“In addition, since I had learned some information [on daily life], there were some adjustments and changes in terms of my lifestyle. For example, if I learned some information that may be good for my disease, then I will try to follow that. Just like the ‘Huangqi,’ a kind of Chinese herb. Since the head nurse told me that Huangqi can accelerate qi-blood circulation and may be good for my disease, I put a piece of Huangqi in boiling water and drink the water every day.”* (P6, Male, 48-year-old, Hepatic cancer, Stage IV)

### Being more prepared for the caregiving role

Being prepared for what lies ahead is an important aspect of feeling in control and relieving uncertainty. Some of the caregivers in this study reported that gaining adequate information would enable them to feel more prepared for their caregiving role:

*“Of course, information on diet is very helpful for us. For patients, actually they need not only medical treatment but also diet, the food therapy. As the patient’s family members, knowing more information on diet will enable us to take care of the patient better at home. We will know what to do and how to take care of him.”* (C8, Daughter, 33-year-old, Patient with hepatic carcinoma)

Adequate information that enabled them to be psychologically prepared for the death of the patient was also mentioned by the caregivers:

*“Learning more information, I think it would be helpful for us as we would feel calmed down when we meet some situations in the future. Learning more, I feel it would help us have preparation, such as the disease condition. Actually, I have learned that this kind of disease cannot be cured, and he will die someday. Learning this can help us have a psychological preparation for the patient’s leaving. We could adjust ourselves in advance to accept the result, which may enable us to feel not too saddened when the patient passes away.”* (C14, Son, 32-year-old, Patient with gastric cancer)

## Discussion

Although ‘silence as virtue’ is valued in Chinese culture, the advanced cancer patients and caregivers needed more ‘communication,’ particularly with healthcare professionals, to get more reliable information, which indicated that ‘silence as virtue’ was not the case for the population with health threats. The significant information needs of the patients may also have been attributed to the relatively young age of the participants (most were younger than 55 years old) as young cancer patients were found to have greater information needs than older ones [33]. Information has been demonstrated to play a critical role in individuals’ efforts to cope with cancer [34] as the diagnosis of cancer is a significant trauma for both patients and family members [35]. Patients and their families often feel stressed about making medical decisions due to the severity and life-limiting nature of cancer [36]. In accordance with previous studies, the Chinese patients and caregivers in this study also indicated that adequate and appropriate information provision may increase their involvement in decision-making and result in greater satisfaction with treatment options [37, 38]. Moreover, the patients and caregivers in this study perceived that appropriate and adequate information could increase their hope for survival and reduce their psychological burden. This study finding was consistent with that of some previous studies, which showed that providing appropriate information that was congruent with the patients’ and family members’ needs could reduce their mood disturbance such as anxiety [9]. Some types of information or an inappropriate amount of information can indeed increase individuals’ mental distress and feelings of hopelessness [39]. Too much information may also render patients unable to cope with their health threats due to high levels of fear and anxiety [40]. In this study, the view of ‘too much information may increase psychological burden’ was also identified by the patients and caregivers. Thus, how to develop and provide tailored and appropriate information regarding information type and amount to patients and caregivers should be considered. Additionally, the patients and caregivers in this study perceived that having adequate and appropriate information could be a way of improving the patient’s capacity for self-management and a way of increasing a sense of control and certainty for the family members taking care of the patient. These findings were in line with several previous studies, which suggested that patients who did not obtain adequate information had less confidence regarding their ability to manage health-related issues [34]. Patients who are adequately informed about their disease are also better able to maintain a sense of control over their cancer [34], as well as deal with the uncertainty of the disease [41]. Thus, information provision with appropriate types and amounts of information can contribute to improved health competence and better symptom management [34, 42].

The information needs of Chinese advanced cancer patients and their caregivers were both explored in this study, and the types of information that the patients desired to learn were broadly similar to those of the caregivers. This finding was supported by two systematic reviews [43, 44] and suggested that both the patients and the caregivers should be involved in conversations with healthcare professionals such as joint meetings [45]. It was not surprising that the patients and caregivers in this study frequently sought disease and treatment information as most of the patients were still receiving active cancer treatment. Patients at the treatment stage are more interested in exploring their treatment options and learning about the potential treatment effects and side effects associated with the treatment options [43]. TCM was reported as important treatment-related information by both the patients and the caregivers in this study. Given the belief in and popularity of TCM in Chinese culture, using TCM to fight illnesses and maintain health is regarded as a natural part of the cultural practices of Chinese people [16]. Inconsistent with some previous studies [43, 44], information on food therapy was another important unmet information need reported in this study. According to TCM theories, illness is caused by an imbalanced Yin and Yang [46]. A proper diet can nourish ‘Zang Fu’ organs and balance Yin and Yang to maintain people’s health and promote the rehabilitation of the disease [47]. This study finding highlighted that the patients’ and caregivers’ information needs were context-bound, and as such, some culturally sensitive information should be assessed and provided within a certain context. The findings also demonstrated that both the patients and the caregivers desired to optimize the patient’s comfort and deal with the negative emotions of the patient by learning more relevant information. This finding was only partly consistent with some previous studies [43, 44] as the motivation of the caregivers to learn information on psychological adjustment in this study was to cover their own negative emotions in front of the patient rather than to maintain their own mental health. Chinese caregivers are usually patient-centered, and they are concerned more about their patient’s conditions than their own health [48, 49]. Although financial issues emerged as a significant concern for both the patients and the caregivers in this study, very few families expressed that they would choose to withdraw treatment for their patient due to financial strains. This might have been because Chinese caregivers often have the attitude of treating the patient’s disease at all costs [50].

Both the patients and the caregivers viewed doctors as the most reliable source of health information. Such a strong reliance on healthcare professionals may be a reflection of strong interpersonally oriented cultural values [51] as many studies have shown that Asian patients often prefer interpersonal information sources [52, 53] rather than other sources. As the preferred information providers, healthcare professionals also need to recognize the importance of learning patients’ health information literacy, that is, the patients’ capacity to access, process, and understand the information they need [54]. In this study, most of the patients had a primary school education background or below; thus, a combination of written information with additional visual and auditory illustrations and materials be considered to improve the patients’ understanding, as individuals with basic education or below usually have a significantly higher risk of inadequate health information literacy [55]. Moreover, healthcare professionals need to recognize the value of having separate conversations with patients and their caregivers, as some caregivers in this study mentioned that they preferred to hold back unpleasant information from the patient for the purpose of protectiveness. However, it was not uncommon that the patients wanted to be told the truth [56]. Some of the advanced cancer patients in this study emphasized that they hoped to be respected, and that any separate discussions between the healthcare professionals and their family members should be conducted based on their permission first [57]. Holding back information from patients was therefore considered unethical [57]. In other words, it was an ethical dilemma for the healthcare professionals regarding how to respond to the family members’ request to hold back information from the patient [58, 59]. Considering that the views of patients and family members regarding truth-telling and patient autonomy are culturally sensitive [43, 56], more studies are needed in the future to explore the perceptions of patients, caregivers, and healthcare professionals regarding the issue of truth-telling in the Chinese context. In addition, consistent with some previous studies [35, 60], the patients’ and caregivers’ preferences for the types of information were not static but changed across the cancer trajectory in this study, which indicated that different types of information should be provided at the appropriate time.

Although healthcare professionals were cited as the ideal information source by both the patients and the caregivers in this study, they were sometimes reluctant to approach healthcare professionals for information, not wanting to occupy too much of their time as they were extremely busy. This finding was supported by previous research [61, 62], which showed that cancer patients were less comfortable asking their physician questions due to their time constraints. Some patients and caregivers in this study held the belief that daily life and diet issues were unimportant issues even though they desired to learn more about them, and they felt that these issues did not deserve healthcare professionals’ limited time. This study did not provide insights into the reason for this perception; however, the high instrumental value and low self-worth placed on authority in Chinese culture could have played a role [51]. ‘Being afraid of facing bad news’ was also a major reason for patients’ and caregivers’ unmet information needs. According to the information-seeking paradigm [63, 64], not all individuals deal with health threats by actively seeking information, and some prefer distraction and avoid receiving too much information. Thus, the differences in individuals’ desire for information should be recognized, as not everyone can benefit from the same amount of information [65]. In this study, family support was sometimes limited for the Chinese caregivers as available family members tended to be a small group due to the one-child policy. The spouse of the patient was usually required to take care of the patient as well as carry out all the domestic tasks, particularly female caregivers. The imbalance between the caregivers’ capabilities and demands undoubtedly imposed heavier burdens on them and subsequently prevented them from having the time or energy to seek information for either the patient or for themselves [19]. Therefore, healthcare professionals should be concerned about insufficient family support experienced by caregivers and proactively assess and provide them with appropriate types and amounts of information.

This study has some limitations. The method of data collection, semi-structured interviews, to some extent may have prevented the participants from expanding on their perceptions and experiences regarding their information needs compared with unstructured interviews. Given that all the recruitment was conducted in the hospital, the perceptions and experiences of patients with unmet information needs in other settings were therefore not captured. Generalizability of the study findings might be another limitation because as a qualitative study, it was specific to a small number of participants within a particular setting, although detailed descriptions of the research context and findings and sufficient and representative extracts (i.e., vivid quotes from the participants) were provided to help readers determine how far the findings could be transferred to other situations.

### Implications for practice and research

To address patients’ and caregivers’ unmet information needs, in addition to increasing the amount of information, the underlying reasons for their unmet information needs should be assessed and considered, such as their emotional status and knowledge and beliefs. Regarding specific information (e.g., information on prognosis), the amount of information should be flexible as it may have disadvantages for some patients and caregivers. Regarding the format of giving information, written information in combination with adequate illustrations (e.g., figures) and other visual and auditory materials (e.g., video and audio) should be considered to accommodate patients with different levels of health information literacy. Given the limited time and the similarity of the information needs of patients and caregivers, viewing patients and caregivers as a ‘whole unit’ when providing information may be an effective approach. However, the value of having separate conversations with patients and their caregivers should also be recognized, as some caregivers in this study preferred to conceal unpleasant information from the patient. Nevertheless, such separate conversations should be performed with caution, as concealing information from patients and discussing the patient’s disease without permission is considered unethical. Given that truth- telling and patient autonomy are culturally sensitive [43, 56], more studies on patients’, caregivers’, and healthcare professionals’ attitudes toward truth-telling in the Chinese context are worth exploring further.

## Conclusion

In this study, information played a critical role in the Chinese patients’ and caregivers’ efforts to cope with cancer, and they perceived that appropriate information provision could facilitate informed decision-making and greater satisfaction with treatment options, reductions in psychological disturbances, enhanced confidence in and ability of self-management, and the ability to anticipate and prepare for caregiving. The findings from this study can promote healthcare professionals’ understanding in terms of what and how much information, from whom, in what format, and when during the trajectory of cancer Chinese patients and caregivers prefer to receive it.

## Data Availability

The datasets used and/or analyzed in this study are available from the corresponding author upon reasonable request.
